# Pacific community’s perceptions on how to improve uptake of urate-lowering therapy for Pacific gout patients

**DOI:** 10.1186/s12939-025-02465-8

**Published:** 2025-04-02

**Authors:** Samuela Ofanoa, Malakai Ofanoa, Siobhan Tu’akoi, Hinamaha Lutui, Maryann Heather, Felicity Goodyear-Smith

**Affiliations:** 1 Pacific Health Section, University of Auckland, 507, 28 Park Avenue, Grafton, Auckland, 1023 New Zealand; 2 Southpoint Family Doctors, Auckland, New Zealand; 3 Department of General Practice & Primary Health Care, University of Auckland, Auckland, New Zealand

**Keywords:** Pacific, Co-design, Urate-lowering therapy, Education, Access to primary care, Community participation

## Abstract

**Objectives:**

This study aims to explore the views of Pacific people on how to improve the management of urate-lowering therapy among Pacific gout patients in South Auckland.

**Methods:**

The study used the Fa’afaletui model to explore the views on gout management across different perspectives of Pacific community members and health professionals working in health providers with high Pacific patient populations. Three workshops were delivered with the community and health professionals. All data were collected by note-takers and analysed using a general inductive approach. Participants were given the opportunity to review the results of the previous workshop before starting the next one.

**Results:**

The key findings from this study clustered into three key themes: [1] Gout interventions guided by Pacific frameworks and research; [2] a multifaceted approach including education, improved access to treatment and screening for gout, and strategies to help Pacific gout patients remain engaged and adherent; and [3] Pacific gout champions.

**Conclusion:**

This study presents the views of community members of how to improve the uptake of urate-lowering therapy among Pacific gout patients. The community highlighted the need for a multifaceted intervention governed by a Pacific model, addressing access barriers and education for Pacific gout patients.

## Introduction

Gout is a chronic arthritis caused by high serum uric acid levels in the body that crystalise and deposit in the joints over time, resulting in painful gout flares [1]. If untreated, gout can impact on quality of life through recurrent gout flares, joint damage and other health conditions such as cardiovascular disease with concomitant disability and loss of dependence [2, 3].

Globally, there were 41 million cases of gout in 2017, with 1.3 million years of life lost due to disability [4]. Aotearoa, New Zealand (NZ) has the highest incidence rates of gout in the world, with 190 per 100,000 in 2017 [4]. In 2016, Pacific people in NZ, experience the highest burden of gout at a rate of 14%, compared to 9% in Māori and 4% for non-Māori non-Pacific (NMNP) [5, 6]. Pacific people are more likely to experience earlier onset, higher recurrence rate, and to be hospitalised for gout flares [7]. Elevated serum urate is the main risk factor for gout [8]. Genetics, weight and kidney problems are considered to be significant contributing factors, with diet only a small contributor [9, 10].

Taking long-term urate-lowering therapy (ULT), such as allopurinol, can prevent gout flares and lead to considerable social [11] and health benefits [2, 3]. Pacific peoples in NZ receive less regular ULT (35% versus 44%) and are hospitalised at nine times the rate of NMNP [7, 12]. Furthermore, Pacific gout patients are more likely to be prescribed increased non-steroidal anti-inflammatory drugs, which may increase their risk of kidney disease [7]. Despite identification of this under-treatment, regular use of ULT among Pacific remains low, and there is an urgent need to improve their gout management [13].

Barriers to receiving ULT include understanding its importance for gout management; access to health service, including clinic opening hours, sub-optimal titration, and stigma around gout [14–16]. To date, most interventions designed to improve the uptake of allopurinol in NZ used a multifaceted approach [17]. However, the evaluations of these interventions indicated issues with engagement, retention and maintaining Pacific and Māori population groups within the programmes over time, particularly in the earlier stages of design which is vital for reducing inequities in gout health [18]. Therefore, this research aims to describe the approaches and key findings from a co-design research project with two Pacific community groups, on improving the uptake of ULT for Pacific gout patients in South Auckland, NZ.

## Methods

### Study design

This study used a constructivist approach to explore Pacific gout through Pacific communities’ experiences and interactions [19]. This paradigm acknowledges that individuals construct their own realities and understandings of the world, and therefore the Fa’afaletui model, a Samoan framework, was also used in conjunction to uphold Pacific people’s experiences of the world. The Fa’afaletui model promotes the gathering and validation of insights and knowledge from different perspectives: the ‘top of the mountain’, ‘top of the tree’, and the ‘person in the canoe’ [20]. It is underpinned by the Pacific values of vā (relational space) and respect which promotes collective decision-making and recognises that the community are the experts [21].

### Recruitment

**Fig. 1:**
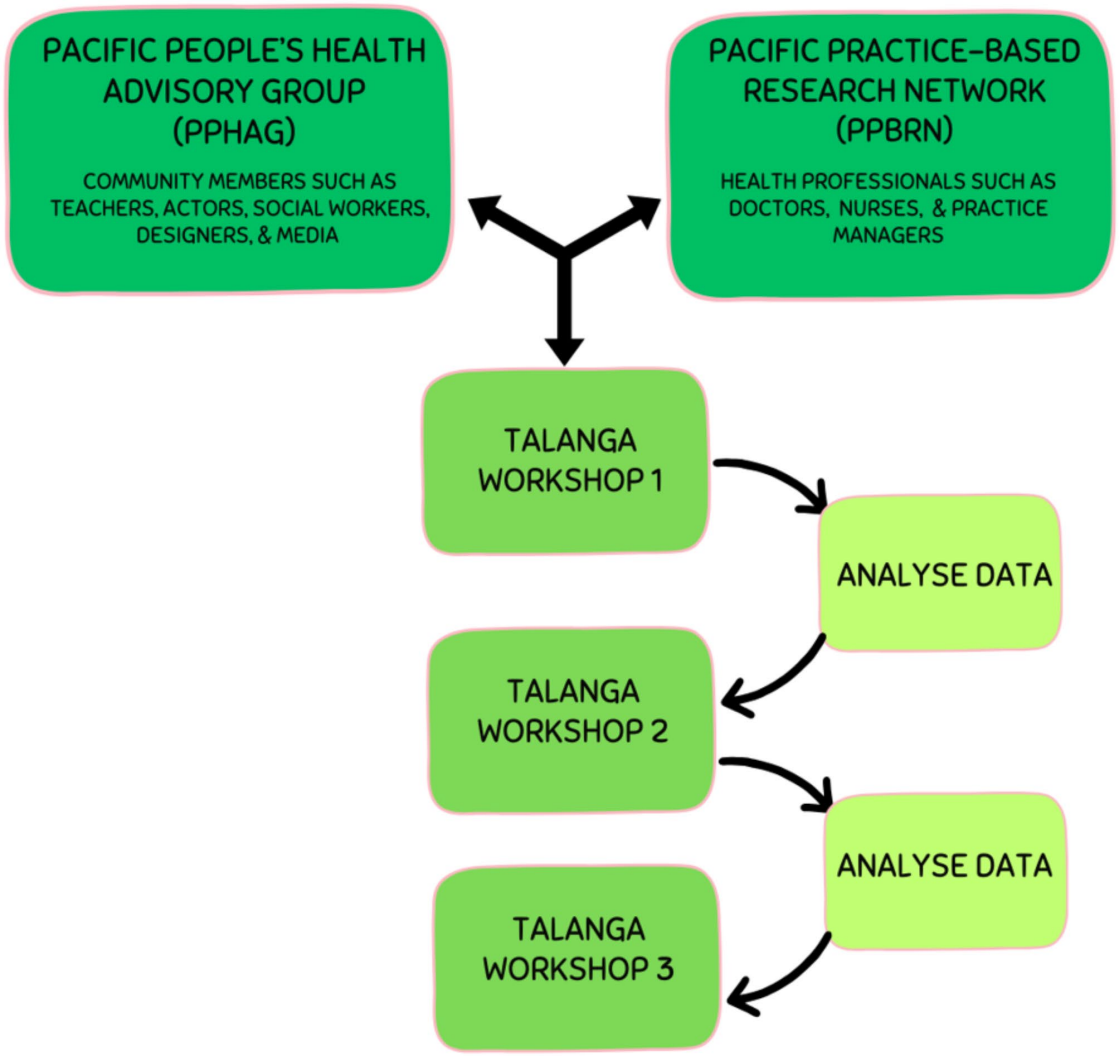
Step by step process of study design with PPHAG and PPBRN

Participants from this study were recruited from PPHAG and PPBRN after providing informed consent (Table 1). The creation of PPHAG and PPBRN has been published elsewhere [22]. In brief, the Pacific People’s Health Advisory Group (PPHAG) is a community- initiated group comprised of South Auckland community members aged between 20 and 80 years, from different Pacific ethnicities and a diverse range of education and social backgrounds (Fig. 1) [22, 23]. The Pacific Practice Based Research Network (PPBRN) was set up through Alliance Health Plus, a Primary Health Organisation, and consists of health professionals from different general practices in South Auckland that cater to high numbers of Pacific patients [24]. Both groups met to discuss important health issues affecting Pacific people in South Auckland, and invited university researchers (MO, FG) to deliver workshops on Pacific methodologies and how to ask a research question. After deliberating and prioritising important health issues, PPHAG and PPBRN identified gout and the uptake of ULT as the most important issue in the South Auckland community [22, 23]. The research team then successfully applied for funding to enable the groups to explore their chosen research question. The number of participants aged between 18 and 80 years who attended each workshop ranged from 12 to 16. Due to contemporaneous COVID-19 restrictions and safety concerns, workshops were delivered online via Zoom. Also, many health professionals in the PPBRN group were unable to participate in the workshops because of COVID-19 commitments.

**Table 1:** Overview of PPHAG and PPBRN members

Characteristic	Workshop 1 (*n* = 16)	Workshop 2 (*n* = 12)	Workshop 3 (*n* = 12)
**Gender**			
**Female**	12 (75%)	9 (75%)	9 (75%)
**Male**	4 (25%)	3 (25%)	3 (25%)
**Gender diverse**	0 (0%)	0 (0%)	0 (0%)
**Ethnicity**			
**Samoan**	10 (63%)	7 (59%)	7 (59%)
**Tongan**	4 (25%)	3 (25%)	3 (25%)
**Niuean**	1 (6.3%)	1 (8%)	1 (8%)
**Other**	1 (6.3%)	1 (8%)	1 (8%)

### Data collection

Three half-day workshops were conducted with the PPHAG and PPBRN utilising Talanga, a Tongan research methodology referring to an interactive conversation with a purpose [25]. All Pacific researchers (S.O, S.T, M.O, M.H and H.L) attended and helped facilitate the three workshops. In workshop one, the research team presented key findings from a stocktake of both international and local interventions which aimed to improve the uptake of ULT in order to explore the community’s views on these approaches [17, 26]. The group was then further broken down and placed into smaller breakout rooms (via Zoom), each consisting of a Pacific research facilitator from the research team, to start discussing and brainstorming ideas on key solutions and ways to improve the uptake of ULT for Pacific communities in South Auckland. Data from each workshop were collected by two researchers (SO, ST) and research assistants via transcribed notes, diagrams, and screenshots by community members and the research team. A similar workshop structure was maintained and delivered in subsequent two workshops.

The main findings from workshop one was presented back to the community at the beginning of the second workshop, providing opportunity for confirmation and feedback of any potential misinterpretations. Workshop two empowered participants to refine the solutions and interventions they brainstormed previously to consider feasibility and cost.

In the third workshop, discussions on the prioritisation of the final community-designed interventions occurred and further feedback ought to choose one intervention moving forward. Participants also provided feedback on the sustainability of the intervention and how to maintain Pacific gout patients in the programme long-term.

### Data analysis

Discussions and notes from the workshops were transcribed by SO and ST and then imported in the NVivo software. Thematic analysis was used to analyse the discussions inductively [27], allowing for patterns, themes and concepts to be derived from raw data [27, 28]. The data was then analysed and interpreted using the six phases of thematic analysis: (1) getting familiar with the data, (2) initial coding, (3) developing themes, (4) reviewing themes, (5) defining and naming themes, (6) writing up findings [27]. Using this process, data and notes from the transcripts were initially coded by two Pacific researchers (SO & ST) and developed into broader themes. These themes were then checked alongside the remaining researchers in the team (MO, FG, HL, MH). These themes were then further reviewed in phase four and due to the co-design nature of this research, these were presented back after each workshop to the community who were key decision-makers in the refinement process and making sure to avoid any misinterpretations of discussions and themes.

## Results

Findings clustered into three overarching themes: Gout interventions driven by Pacific frameworks and research, multi-faceted or multi-layered approach (addressing access barriers, education and maintenance), and the need for Pacific gout champions to lead interventions (see Table 2).

**Table 2:** Themes, sub-themes and illustrative quotes

Theme	Sub-theme	Quotes
Gout interventions guided by Pacific frameworks and research	Name of intervention	*“There are negative connotations of the word ‘coconut’. It may be hard to unwrap the labels around coconut. It might be better to use ‘niu’. Could we come up with an acronym with those three letters?*” Participant 1, PPHAG member
	Nature of intervention	*”[The] coconut [NIU] intervention– outside is really hard and [represents] the challenges; break it open and it has the nourishment [gout medication] and all the experiences we’ve got; the liquid is the sweet that we want to go to [life without gout flares].” Participant 10*, PPHAG member
	Lack of Pacific data	*”There is a lack of data on Pacific gout and pushing this project is the first start. So many other data we use as second hand*,* but first-hand data should be by Pacific for Pacific. It is already happening but not in the most co-ordinated way.”* Participant 5, PPHAG member
Multifaceted/multi-layered approach	Education about gout	*”We need to promote gout heavily in community using billboards*,* social media*,* posters*,* interviews*,* church*,* big Pacific events to increase awareness.”* Participant 2, PPHAG member
	Education about genetic predisposition	*”Studies have shown that we [Pacific] have a genetic predisposition to getting gout.”* Participant 14, PPBRN member
	Education to reduce stigma	*”[We need to] find ways of reducing any stigma about having gout… sharing family members’ stories of how it impacts on family*,* work… once everyone understands this will reduce the stigma.”* Participant 11, PPHAG member
	Education about herbal remedies	*“In my experience people might try herbal remedies but by the time they present to us [doctors]*,* they have severe symptoms. They can’t cope with the pain. These alternatives haven’t worked but instead has increased the risk.*” Participant 15, PPBRN member
	Education via social media	*“A Facebook group page could be used to provide up to date gout information/promotions*,* provide a safe forum for questions to be answered by the members or experts*,* and also be able to celebrate the success stories and achievements… the participants could be sourced from current and long-term patients and families of patients and clinical experts (Pacific doctors / nurses)”* Participant 4, PPHAG member
	Education about allopurinol titration	*“Some of the research that we are seeing is that when Pacific gout patients do go into the clinic*,* they are not receiving optimum titration”* Participant 14, PPBRN member
	Access to appointments	*“People with gout who can’t access healthcare at the right times such as shift-work. That must be a huge factor for not getting care.”* Participant 6, PPHAG member
	Financial barrier to access	*“It depends on the clinic they are at but low-cost clinics are $10 or $19 [consultation fee]. If it’s just gout*,* the script charge is $5 for one prescription*,* and they need this four times a year. Pacific people however have lots of other conditions that need prescriptions and so this adds up.”* Participant 15, PPBRN member
	Pharmacists may provide one-stop-shop	*“Having a person that can deal specifically with gout– follows through with the process with the community. Sometimes it may take days or week to see a doctor and having another person to see you would be great. The doctor could put the patient in touch with them.*” Participant 2, PPHAG member
	Monitoring and testing could be done in the community	*“For monitoring of gout and testing*,* we could consider options that cater to the community’s needs like at the supermarket you could have a test at a mobile van and a nurse could visit patients at their house if needed after.”* Participant 4, PPHAG member
		*“I know that we rely on doing blood tests at the lab*,* but I am not aware of point-of-care testing in primary care clinics… at this stage it’s the lab tests.”* Participant 16, PPBRN member
	Maintaining Pacific gout patients on ULT long-term	*“Attach the education to a community event and have teams of doctors*,* nurses*,* pharmacists*,* educators wearing gout t-shirts and they can walk and talk and bring it to the community level*,* make it a normal conversation at the community level.”* Participant 10, PPHAG member
	Buddy and family support system	*“I think to keep them taking the medication and seeing a GP they should have a buddy in your family to go with you to appointments and monitor medication use. This will also address some of the translation issues.”* Participant 11, PPHAG member
		*“Support for the whole family so the family members are also aware of what gout is and the importance of taking medication daily as a preventative measure.”* Participant 9, PPHAG member
Pacific Gout Champions		*“It’s so good to hear from Pacific doctors and their experience with gout.*” Participant 1, PPHAG member
		*“This can be Pacific led - Pacific doctors*,* nurses*,* patients…and also using high profile Pasifika peoples as ‘Gout & About’ Ambassadors.”* Participant 5, PPHAG member

### Gout interventions guided by Pacific frameworks and research

The community decided that a Pacific model and name was needed as an underlying framework to drive a Pacific gout intervention. They highlighted that the model should enable the intervention to encompass all aspects of a Pacific gout patient’s health and encompass the diversity of the Pacific, stating, “We need a title of the project that relates to us Pacific people” (Participant 10, PPHAG member). This would create a sense of ownership. Although the term ‘coconut’ was suggested as an intervention name, participants acknowledged the negative connotations related to historical discrimination experienced by Pacific people associated with the name. *Niu*’ was decided upon as it has a common translation for coconut’ across most Pacific nations. The community members reached consensus that NIU could be an acronym that stood for ‘***N****esians****I****mproving****U****rate-lowering therapy’* for Pacific gout patients in South Auckland. The term ‘Nesian’ includes all Pacific belonging to Pacific cultures across Micronesia, Melanesia, and Polynesia.

One participant indicated the importance of building the knowledge base of Pacific health data and ensuring this is used by Pacific for Pacific gout patients to drive best practice (Participant 5, PPHAG member).

### A multifaceted/multi-layered approach

Community members spoke about the “complex realities of living with gout” (Participant 1, PPHAG member) in South Auckland. Most barriers highlighted could not be addressed by a single intervention. One participant shared, “A multifaceted approach is needed, especially with the complex realities that Pacific gout patients experience” (Participant 4, PPHAG member). Participants noted that interventions needed several layers to reach diverse Pacific population groups and that there was a need for “Multi layered approaches focussing on education, services and adherence” (Participant 7, PPHAG members).

### Education

Community groups emphasised the importance of education for improving the uptake of ULT among Pacific gout patients in South Auckland. Despite a range of existing educational strategies, “still need more education and a preventative approach” (Participant 9, PPHAG member) as gaps in awareness remain regarding gout, its risk factors, and medications. Participants recommended focussing on primary prevention strategies whereby gout information could be disseminated via wide-scale campaigns and delivered annually, to maintain and reinforce understanding. The groups asserted that campaigns should be simple, culturally appropriate and have different versions to acknowledge diverse cultures, backgrounds, and age-groups.

Education to identify gout symptoms early and prevent the condition worsening was also discussed. Focus on contributing factors such as alcohol and diet place the onus on the Pacific patients often resulting in stigma, shame, and self-blame. Acknowledging this, the community indicated that education could empower Pacific people with gout to share their own experiences and normalise seeking treatment. The initial engagement between Pacific gout patients and health professionals was viewed as vital to initiating understanding gout and medications, with community members suggesting health professionals could use resources that are more “visual, physical, simple and relevant” (Participant, 11 PPHAG member). They discussed that creating a Pacific gout community page could provide access to support from health professionals, other Pacific gout patients and organisations, and ensure they “don’t think that they’re the only one’s suffering” (Participants 4, PPHAG member). This could also address other common issues such as sharing medications between family members, which one PPBRN member discussed as being “prevalent among families and friends and this is a consequence of all the education and access barriers that Pacific gout patients experience”. PPBRN members (Participant 15 & 16) had experienced Pacific patients seeking alternative treatments which exacerbated their gout symptoms.

Additionally, PPBRN members highlighted the importance of focussing education initiatives towards health professionals and the system, asking “what about educating the health professionals?”, and indicating that increasing understanding of the realities that Pacific gout patients face and standardising how gout information is delivered could avoid confusion and misinterpretation.

### Access

Community members reported barriers to accessing healthcare such as limited appointment times, inaccessible clinic opening hours, and significant treatment costs, particularly when combined with other comorbidities. PPBRN members described how Pacific patients often seek treatment at hospital, which may address the symptoms but not the underlying risk factors for gout, with follow-up difficult post-discharge.

In response to these barriers, community members highlighted the need for alternative points of access by utilising other health professionals. PPBRN general practitioner members agreed that the role of nurses, pharmacists and other health professionals has evolved over time and that they are capable of delivering gout management services, “Pharmacists’ jobs have changed a lot, and they want to be involved in patient care and education to ensure patients understand” (PPBRN members). Participants agreed that patients should be at the centre of healthcare systems and be able to choose where is most suitable for accessing their gout management.

Access to screening was also identified, with community members expressing that Pacific people often take advice from family members rather than seeing a health professional– “it’s very common being diagnosed by an uncle” (Participant 11, PPHAG member). Several community members suggested the need to bring screening into more convenient community spaces, allowing for easier follow-up and monitoring of urate levels. All PPBRN members agreed but were uncertain about the validity and effectiveness of point-of-care testing in the community. There was a general agreement around the benefits of Pacific providers to conducting screenings, but the limited Pacific workforce was noted, with one member identifying “a lack of Pacific pharmacists, less than a dozen in the entire country” (Participant 15, PPBRN members).

### Maintaining Pacific gout patients on ULT long-term

A common sub-theme emphasised by all PPHAG and PPBRN members was the challenge of long-term ULT maintenance. Community members spoke about how interventions may be effective, but often only run for a short period of time with access barriers re-experienced after programmes end, “After a few months when we are better we go back to the GP and that is where we stop taking the medications” (Participant 4, PPHAG member). A PPBRN member agreed “Yes, people do come back to the clinic when they don’t finish programmes or when programmes finish. This is often when they get pain flare-ups” (Participant 15). Participants emphasised the need for ongoing information about gout, which could be achieved by annual events such as a ‘gout walk’ or incorporating gout topics into the national Pacific language weeks (Participant 10, PPHAG members). Collaborations and sponsorships with key organisations were seen as critical for sustainability, alongside regular evaluations to measure effectiveness.

The importance of involving the entire family was emphasised. A system whereby gout patients identify a family member as a buddy to help them monitor their medications and make appointments, was seen as a way to increase accountability for regular clinic attendance, spreading awareness and addressing language challenges. Simple reminders such as texts and follow-up phone calls to patients and families could help significantly. As an example, members suggested “Fridge magnets or stickers to remind you to take your medication when you open the fridge or even mugs with Pacific messages about taking your medication in different languages” (Participant 11, PPHAG member). Celebrating achievements and progress with taking medications was also proposed, “We could give out healthy gifts or progress badges and after three months you get this or after 12 months you get this” (Participant 12, PPHAG member).

### Pacific gout champions

The community emphasised that interventions needed to be Pacific-led, using ‘Gout Champions’ as ambassadors to increase awareness and normalise conversations about seeking treatment. Pacific gout champions were seen as vital to foster relationships with the community to ensure on-going engagement and trust with the health and education services being promoted. Champions suggested included Pacific health professionals, high-profile Pacific people and Pacific community members affected by gout.

*“It’s so good to hear from Pacific doctors and their experience with gout…” Participant 2*,* PPHAG member.*

*“This can be Pacific led– Pacific doctors*,* nurses*,* patients…and also using high profile Pasifika peoples as ‘Gout & About’ Ambassadors.” Participant 4*,* PPHAG member.*

## Discussion

This study explored Pacific community members’ and health professional views on how to improve the uptake of ULT for Pacific gout patients in South Auckland. This adds to the paucity of research in this area, as previous qualitative studies that have included Pacific participants have not reported Pacific-specific findings [29–31], while other studies report on Pacific gout prevalence or trends [14, 32–34], impacts of gout [35] and genetics [36–39].

The community members of the present study identified the need for a Pacific framework and name to drive the Pacific gout intervention. It was important to encompass all facets of a gout patient’s health and to create a sense of ownership and relatability for Pacific gout patients. One NZ study [40] had reported distinct worldviews when examining Māori whānau/community experiences to barriers and enablers to gout management. This study found two emerging domains of Māori and Western perspectives and the weaving of these solutions to shift power, enabling community members to be decision makers and leaders in designing gout interventions. Pacific community members in our study suggested that Pacific gout champions could help lead interventions and foster relationships with the community, to ensure on-going engagement and trust with the services and education being promoted.

A key theme was continued lack of awareness and understanding of gout, its risk factors/triggers, and medications among the wider community. The knowledge gap was exacerbated by the absence of Pacific-specific gout data and research that could drive best practice education strategies. Similar to a previous NZ study [40], our community members suggested that a national media campaign using social media could raise widespread awareness in the population and reduce stigma. Stigma related to gout is commonly linked with a lack of understanding of the contributing factors to increased gout flares, particularly with diet, where high consumption of alcohol and seafood is said to be the main cause [40–43].

For Pacific gout patients in particular, participants discussed the challenges of understanding diagnoses, medications, management, and the seriousness of the disease. A qualitative study in Australia of 10 Pacific men aged between 16 and 55 years reported that most participants experienced a lack of information provided by health professionals after a gout diagnosis [31]. Community members in the present study suggested that creating a gout community group could foster a safe discussion forum with experts providing educational support on gout management practices, and ambassadors talking about their gout experiences. This could create a culture which normalises conversations about gout and seeking long-term treatment. Another NZ study [15] exploring patient feedback on a gout intervention programme reported that out of the 16 patients who completed the programme, 13 were still using allopurinol after the programme but only eight could remember that taking allopurinol needed to be long term. This suggests to not only provide appropriate education early, but also to have ongoing information after programmes end to reinforce gout health literacy.

Barriers to accessing gout treatment have been well established in previous research and have shown to exacerbate inequities in gout health [14, 18, 31, 32, 40]. Common factors restricting access to gout treatment are appointment times, having to wait days or weeks to see a general practitioner (GP), and patients’ employment hours preventing access to GPs during their opening hours [14, 31, 40]. PPBRN members reported that although the costs to patients for gout prescriptions, may seem manageable, when compounded with the treatments and medications needed for other chronic health conditions, costs can accumulate and cause a financial burden for Pacific gout patients. Coleman and colleagues [31] reported that the debilitating effects of gout symptoms and flares on a person may result on the loss of their independence, hence family members may miss work to support them which then worsens their financial stress. Current health systems and policies need to respond to these structural issues, particularly when they continue to inhibit optimal gout care in population groups such Māori and Pacific who are most affected by gout and other comorbidities.

In response to addressing access barriers, Pacific participants in this study reported the need for alternative screening and treatment access points. The community suggested that using pharmacists to receive their gout treatment could avoid long waiting times for doctor appointments and blood tests. Over the years, there has been an increase in successful pharmacist-based interventions around gout in NZ [17, 26]. Both the Own My Gout and Gout Stop programmes are multifaceted interventions in NZ that use pharmacists to improve equity in initial access to education, treatment and management of gout [18]. An evaluation report showed that both interventions successfully increased access to enrolment (particularly for men and younger groups) and reduced urate levels of patients who successfully completed the programmes [15, 18]. When reviewing these interventions, participants in this study identified that often when such programmes end, patients experience their original access barriers when transferred back to their GPs. The importance of maintaining Pacific gout patients on treatments long-term was identified as critical. A recent NZ study [32] showed that although rates of dispensing of allopurinol was high after discharge of gout patients from the hospital, the rates of regular dispensing was low (38%). More effort is needed during follow-up after hospitalisation to ensure patients maintain ongoing dispensing or treatment, particularly in GP care.

A possible limitation of this research is that our PPHAG community group are primarily Polynesian (who are the vast majority of Pacific) and thus lacks perspectives from Melanesian and Micronesian ethnic groups. Community members reside in South Auckland; therefore these experiences may differ from Pacific groups in other areas in NZ. Additionally, because workshops took place while COVID-19 was still prevalent in NZ, health professionals had higher work demands, hence many PPBRN members were unable to attend. A key strength of this research is the use of the Fa’afaletui model to explore both community and health professionals’ perspectives. While views from both PPHAG community members and PPBRN health professionals were generally in agreement, certain perspectives not addressed by the community were often supplemented by the health professionals. This is the strength of the Fa’afaletui approach. Another strength is the co-design partnership for this research that started more than five years ago, allowing time to build strong relationships. PPHAG and PPBRN were formed prior to university partners coming on board and came up with the research question themselves, thus are the true owners and leaders of this research. Developing, implementing, and evaluating this intervention will assess its effectiveness in improving Pacific health outcomes.

## Conclusion

This study explores the views of Pacific communities for improving the uptake of ULT among Pacific gout patients. A multifaceted approach underpinned by a Pacific framework is vital to develop a ‘by Pacific, for Pacific’ intervention. Given that Pacific and Māori are over-represented in gout statistics, current interventions and systems should incorporate their perspectives to more effectively reduce gout inequities in NZ. Involvement of the community in developing a research question to improve health outcomes for their population and then answering it facilitates buy-in and ownership of the final intervention.

## Data Availability

No datasets were generated or analysed during the current study.
